# Experimental Diagnosis on Combustion Characteristic of Shock Wave Focusing Initiation Engine

**DOI:** 10.3390/e24071007

**Published:** 2022-07-21

**Authors:** Shida Xu, Feilong Song, Xin Chen, Hesong Zhang, Xingkui Yang, Jianping Zhou

**Affiliations:** Science and Technology on Plasma Dynamics Laboratory, Air Force Engineering University, Xi’an 710038, China; xushida950625@163.com (S.X.); chenxin7605@aliyun.com (X.C.); zhang709152164@163.com (H.Z.); yangxingkuirde@163.com (X.Y.); zhoujianpingrde@163.com (J.Z.)

**Keywords:** shock wave focusing, combustion mode, pyrolysis, nonlinear time series analysis, pressure utilization

## Abstract

A shock wave focusing initiation engine was assembled and tested in an experimental program. The effective pyrolysis rate of the pre-combustor was evaluated over a range of supplementary fuel ratio in this paper. Results highlight two operational modes of the resonant cavity: (1) pulsating combustion mode, (2) stable combustion mode. The appearance of the two combustion modes is jointly affected by the flow and the structural characteristic value of the combustion chamber. This paper uses images, time-frequency analysis, and nonlinear time series analysis methods to identify and distinguish these two combustion modes. It is believed that the interaction between the combustion chamber and the supply plenum is the probable reason for different combustion modes. The experiment has found that structural parameters and import flow parameters have an impact on the initiation of the combustion chamber. Increasing the injection pressure can appropriately broaden the fuel-rich boundary of initiation. Low equivalence ratio and high injection pressure can also appropriately increase the combustion working frequency in a small range. From the perspective of pressure utilization, under the premise of ensuring successful initiation, injection pressure should not be too high.

## 1. Introduction

The thermal cycle efficiency of a detonation engine is higher than that of a traditional turbine engine due to the process of approximate isometric combustion [1,2,3,4]. The advantages of a constant volume combustion cycle as compared to constant pressure combustion in terms of thermodynamic efficiency has focused the search for advanced propulsion on detonation engines [5]. At present, most of the kerosene-based pulse detonation engines have problems such as the DDT (deflagration to detonation transition) process [6] and low working frequency [7]. Levin et al. [8] innovatively proposed a valveless high-frequency 2-stage pulse detonation engine scheme in 2001. In this scheme, kerosene was decomposed into active components by pre-combustion pyrolysis. The high-speed air-fuel mixture (AFM) flowed into the hemispherical resonant cavity through the annular slot, and the fuel could be initiated (or detonated) by the high-temperature and pressure region generated by the shock wave focusing. Compared with the traditional pulse detonation engine, the working frequency of this engine theoretically will not be limited by the ignitor and valve frequency, the structure is more compact, and it is not easy to be effected by the external environment [9]. For this type of shock wave focusing detonation combustor, Levin et al. [10,11,12] also carried out a large number of experiments and numerical simulations on blow-down shock wave focusing combustion using the acetylene-air mixture as fuel. The flow field characteristics, thrust, and specific impulse characteristics inside the engine cavity, and the spectrum characteristics of shock wave focusing combustion under different structures were studied thoroughly. The high frequency detonation combustion of thousands Hz was obtained through experiment and simulation research. Keith R et al. [13] also carried out a large number of experiments of the above valveless high-frequency 2-stage pulse detonation engine, and studied the combustion wave characteristics of methane, ethylene, and kerosene fuels, but failed to determine a detonation wave. It is worth noting that both Levin and Keith R have found that there are two combustion modes in this shock wave focusing combustor, but the cause of this phenomenon has not been studied in detail. Smirnov et al. [14,15,16,17] conducted a lot of numerical and experimental investigations of detonation initiation in hydrogen-air mixture due to the focusing of a shock wave reflected inside a wedge. Kinetic schemes and turbulence models were also improved based on a comparison of numerical and experimental results. In their research, four different scenarios (no ignition case, strong initiation case, soft ignition case, and deflagration to detonation transition behind reflected shock wave case) were distinguished. It was found that these different scenarios were all dependent on the incident shock wave intensity. Gray et al. [18] tested a shock wave focusing geometry which exhibited a very short DDT distance for detonation initiation. The pressure evolution was measured at various locations in the detonation tube. Based on these data, a numerical simulation governed by the reactive, compressible Navier-Stokes equations is adapted by means of an adjoint-based data assimilation, where Arrhenius and diffusion parameters are adjusted. Gelfand et al. [19] experimentally investigated the detonation and deflagration initiation under focusing conditions on a lean hydrogen-air mixture. Two-dimensional wedges (53° and 90°), semi-cylinder and parabola, were used as the focusing elements. The peculiarities of mild and strong ignition inside the reflector cavity were visualized. It was also found that mild ignition inside the reflector cavity can lead to detonation initiation outside the cavity. Numerical and experimental studies were also conducted by Zhang et al. [20] to understand the ignition behavior in a stoichiometric methane-oxygen-argon mixture due to shock wave reflection from a variety of shapes, including planar, 60° and 90° conical and hemispherical reflectors. Two ignition modes (namely, weak ignition and strong ignition) were found, which were highly dependent on the incident shock wave velocity. A two-dimensional numerical investigation was carried out by Chen et al. [21] for hydrogen-air mixtures. Toroidal shock wave formation and detonation initiation by shock wave focusing were analyzed. It was found that one possible mechanism of rapid detonation initiation is that shock waves produced by two explosions can compress premixed gas rapidly and accelerate the deflagration to detonation transition. Simulations of initiation by shock focusing with different hydrogen concentrations were conducted by Yang et al. [22]. The results showed that high hydrogen concentration on initiation was significantly effective in accelerating the attenuation of over driven detonation, which was conducive to initiating a quick and stable detonation initiation.

In general, the single shock wave focusing initiation process and mechanism of gaseous fuel have been widely studied by means of experiments and simulations. However, the study of physical process in multi-cycle shock wave focusing initiation in a flowing state is insufficient. In addition, the shock wave focusing initiation characteristics of kerosene and other liquid fuels with aviation power application potential also need to be further studied.

Our research group has been working on a shock wave focusing mechanism [23,24,25] and kerosene pre-combustion cracking components control [26,27,28] research for some time. In this paper, the experimental diagnosis of multi-cycle shock wave focusing combustion characteristics of RP-3 aviation kerosene pre-combustion pyrolysis products was carried out. The influence of import parameters on different combustion modes as well as amplitude and frequency characteristics of the combustion were also revealed in this study. This investigation could guide the practical application of this type of two-stage pulse detonation engine in further research.

## 2. Experimental Facility and Methodology

### 2.1. Experimental Facility

**Figure 1:**
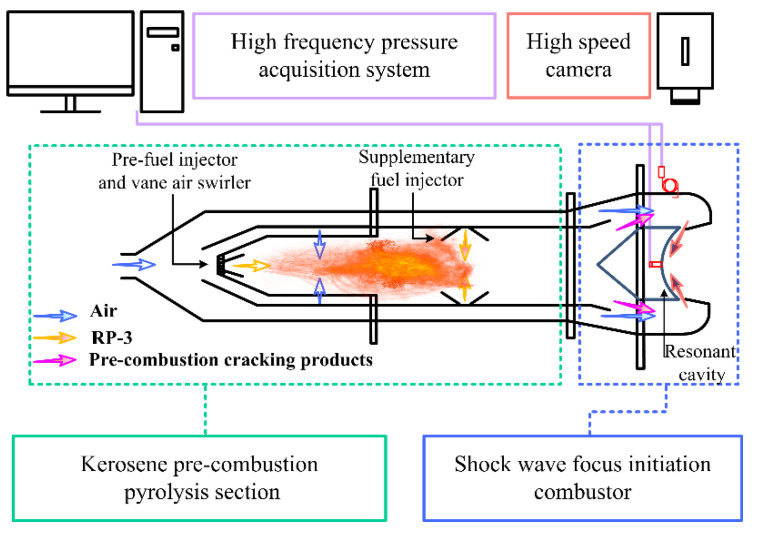
The schematic diagram of the two-stage detonation experiment device.

The schematic diagram of the shock wave focusing initiation (two-stage pulse detonation) experiment device is shown in Figure 1. The experimental device adopts a 2-stage combustion mode.

In the first stage, partial kerosene is pre-burned in a swirl combustion chamber under the equivalence ratio of 0.9 to generate a high-temperature environment. The secondary supplementary kerosene injected is evaporated and cracked to produce highly active components and kerosene vapor that are easier to detonate. In the pre-combustion chamber, the pre-combustion kerosene flow is controlled by an automatic flow regulating valve to keep it constant at about 9 g/s and the pre-combustion air flow is around 145 g/s, which is controlled by a sonic nozzle and calculated upstream pressure. The supplementary kerosene flow can be changed by adjusting the pressurized nitrogen pressure in the fuel tank. The range of supplementary fuel ratio (supplementary kerosene flow/pre-combustion kerosene flow) is changed between 5 and 12. In the second stage, the shock wave of the premixed fuel and air are focused in a resonator to burn or detonate. The first stage pre-combustion pyrolysis product is mixed with the second stream of air from the outer duct. The air flow can also be changed by adjusting the pressure upstream of the sonic nozzle in the main air line. Then the mixture is injected into the resonant cavity at the angle of 15 degrees through a 4 mm or 8 mm width annular slot. The annular slot width is changed by installing gaskets between the two flanges. Driven by the pressure difference, the high-speed airflow accelerates continuously in the convergent channel. A strong shock wave is formed when the high-speed airflow leaves the annular slot, which then can reflect and focus in the resonant cavity to form a local high-temperature and high-pressure region to initiate the air-fuel mixture. The resonator adopts a partial spherical configuration. Compared with the 70 mm diameter resonator in the literature [11,12], the diameter of the resonator selected in this paper is 90 mm and the depth is 23.6 mm, in order to provide a larger thrust wall area.

### 2.2. Measurement Methodology

The pressure at the bottom of the resonator and the injection pressure in front of the annular slot were extracted by the capillary at the bottom of the resonator and the pressure probe in front of the annular slot respectively. The corresponding pressure was detected by static pressure sensor. This measurement method can effectively avoid damage to the sensors as a result of high-temperature gas, but it will also partially weaken the pressure signal amplitude. The pressure signals were collected and processed by the Ni data acquisition system (PXI Express). At the same time, to observe the experimental phenomena more intuitively, a high-speed camera was used to shoot the exit flame of the shock wave focusing initiation combustor. Combined with the pressure signal, the working state of this combustor was analyzed comprehensively. The model of this high-speed camera is Phantom-v2512. In the experiment, the camera parameters were set as follows: resolution 1024 × 768, exposure time 98.49 μs, and shooting frame rate 10,000 fps. The pyrolysis products were obtained by sampling probe and analyzed by the Agilent 7890B gas chromatograph (RSD < 0.008%). The gas chromatograph adopts a three-channel refinery gas program. After being separated by molecular-sieve capillary columns, C_1_–C_5_ hydrocarbon compounds were tested by a flame ionization detector channel and the hydrogen was specifically detected by a thermal conductivity detector channel.

### 2.3. Experimental Conditions

To characterize the parameters of different structures, the structural characteristic value is introduced as follows [8]:

(1) $$q=\frac{S_{C}}{S_{TS}}$$

In Formula (1), $S_{C}$ (mm^2^) is the exit area of the resonant cavity and $S_{TS}$ (mm^2^) is the injection area of the annular slot. The ratio reflects the overall structural characteristics of the combustor. Keeping the exit diameter of the resonant cavity at 90 mm unchanged, the structural characteristic value of the combustor is changed by adjusting the width of the annular slot. When the width of the annular slot is 4 mm, the structural characteristic value is 5.63. When the width is adjusted to 8 mm, the structural characteristic value is 2.81.

Based on previous research results [23,24,25], the experimental conditions are listed in Table 1. In order to investigate the impact of structural parameters and import flow parameters on the shock wave focusing initiation characteristics of the combustion chamber, the experiments were conducted under two types of annular slot width (4 mm and 8 mm), two flows (1.38 kg/s and 1.78 kg/s or so), and three operation equivalence ratios (0.6, 0.8, 1.0 or so) conditions. The experiment controlled the overall equivalence ratio and pressure by changing the flow of fuel and air.

## 3. Experimental Results and Analysis

### 3.1. Composition Analysis of Pre-Combustion Pyrolysis Products

**Figure 2:**
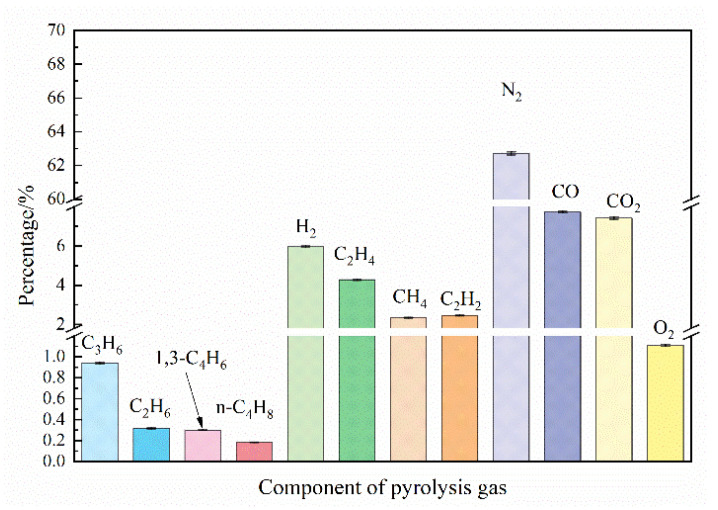
The main components of pyrolysis gas distribution under typical working conditions (working Con. 3).

The main components of distribution of pyrolysis products under a typical working condition (Con. 3) is shown in Figure 2. According to the analysis results of the gas chromatograph, 5.9% hydrogen, 4.2% acetylene, 4.4% methane, 7.7% carbon monoxide, and other small molecular compounds that can participate in detonation combustion can be produced by pre-combustion and pyrolysis of RP-3 aviation kerosene. Table 2 [29] lists the detonation cell size and critical ignition energy of hydrogen, methane, ethylene, and acetylene, as well as the cell size of carbon monoxide mixed with the above four components in different proportions. The test results of pyrolysis gas fully verified that kerosene can be cracked into gaseous components that are easy to detonate, thus greatly reducing the detonation cell size and critical ignition energy of kerosene/air mixture, and greatly improving the initiation and detonation performance of macromolecular kerosene fuel [30].

The pyrolysis products, under different supplementary fuel ratios, were collected and analyzed, and the carbon-based characterization effective pyrolysis rate [28] was used to analyze the secondary supplementary fuel pyrolysis efficiency under different operational conditions. It was found that with the increase of supplementary fuel ratio, the carbon-based characterization effective pyrolysis rate was significantly reduced. The pyrolysis rate decreased from 54.6% at the supplementary fuel ratio of 5.49 to only 20.8% at the supplementary fuel ratio of 12.73. This is a result of the pre-combustion fuel flow and the air flow being kept relatively constant during the experiment, thus the heat generated by the pre-combustion was relatively unchanged. However, as the secondary supplementary fuel flow increased, more heat was used for the evaporation and gasification of sprayed liquid kerosene, which led to a reduction in the heat used for kerosene thermal pyrolysis, which in turn led to an overall decrease in the pyrolysis efficiency of the second supplementary fuel. Therefore, when considering the supplementary fuel ratio, on the one hand, it is necessary to consider the effective pyrolysis rate to ensure the processed fuel activity. On the other hand, it is necessary to minimize the proportion of pre-combustion fuel in the total fuel supply, thereby increasing the proportion of fuel used for detonation combustion.

### 3.2. Shock Wave Focusing Combustion Mode Identification

In the experiment, the influences of different structural characteristics on shock wave focusing initiation were compared by changing the width of the annular slot. The airflow and the corresponding kerosene flow were also changed to compare the combustion modes of shock wave focusing initiation under different import flows and different equivalent ratios. According to the measured combustion wave pressure signal at the bottom of the resonant cavity and the observed experimental phenomena, the influencing factors of the successful shock wave focusing initiation were found. At the same time, after the successful initiation, due to different operational conditions, there existed two different combustion modes named stable combustion mode and pulsating combustion mode.

**Figure 3:**
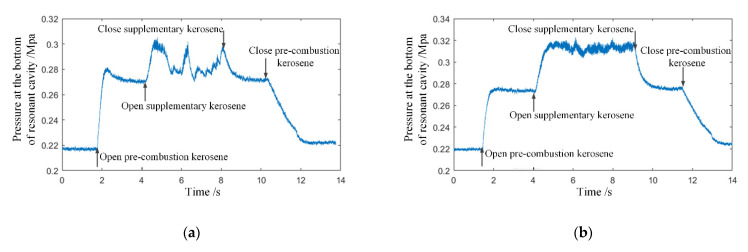
The pressure signal change of successful initiation and failed initiation. (**a**) Failed initiation (12–45), (**b**) successful initiation (12–56).

**Figure 4:**
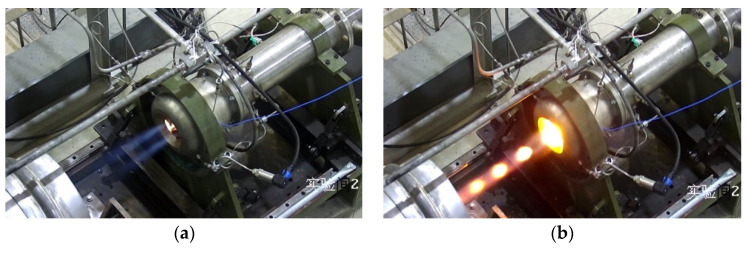
The experimental scenes of successful initiation and failed initiation. (**a**) Failed initiation (12–45), (**b**) successful initiation (12–56).

Firstly, the successful and failed initiation operational conditions were analyzed. By analyzing the pressure signals at the bottom of the cavity and the experimental images, it was found that the small flow, wide annular slot, and high equivalence ratio were the main reasons for the failure of shock wave focusing initiation. The success or failure of the initiation could be judged by the characteristics of the pressure signal change at the bottom of the cavity. Figure 3a shows the typical pressure signal of failure initiation under condition 8. After the supplementary kerosene was injected, the pressure signal at the bottom of the cavity increased sharply, but did not maintain for some time, and rapidly dropped to the level without kerosene supply. Figure 3b is the typical pressure signal of successful initiation under condition 10. It can be seen that the pressure at the bottom of the cavity rapidly increased with large pressure fluctuation after supplementary kerosene was injected. In the case of initiation failure, there existed a large amount of kerosene vapor at the outlet of the resonant cavity and no bright flame appeared. The phenomenon of successful initiation was very obvious. A bright yellow-white flame could be observed at the exit of the resonant cavity, accompanied by a Mach disk, as shown in Figure 4.

The wide annular slot and small flow will lead to the drop of injection pressure, the shock wave focusing intensity will be reduced, and therefore lead to insufficient focusing pressure and temperature for initiation [25]. Moreover, the high equivalence ratio will also lead to failed initiation. On the one hand, high equivalence ratio (corresponding to high supplementary fuel ratio) will lead to insufficient pre-combustion pyrolysis, poor fuel activity as discussed in the above section, and the threshold of required initiation energy will increase [28]. On the other hand, more liquid kerosene without full evaporation can also absorb and reduce the ignition temperature in the shock wave focusing region. Each of these circumstances eventually lead to the failure of initiation. However, increasing the airflow and reducing the width of the annular slot increases the probability of successful initiation due to the increase of shock wave intensity. Meanwhile, a higher injection pressure before the slot results in a wider the initiation equivalence ratio boundary.

**Figure 5:**
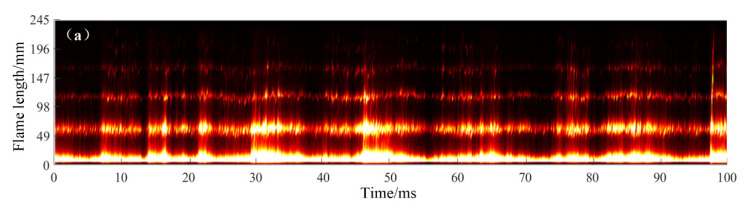

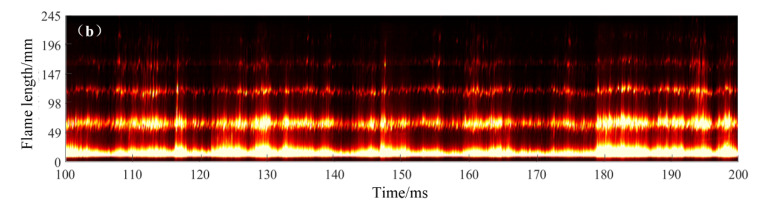
Stable combustion mode tail flame distribution over time (15–18). (**a**) Tail flame distribution over 0–100 ms, (**b**) tail flame distribution over 100–200 ms.

**Figure 6:**
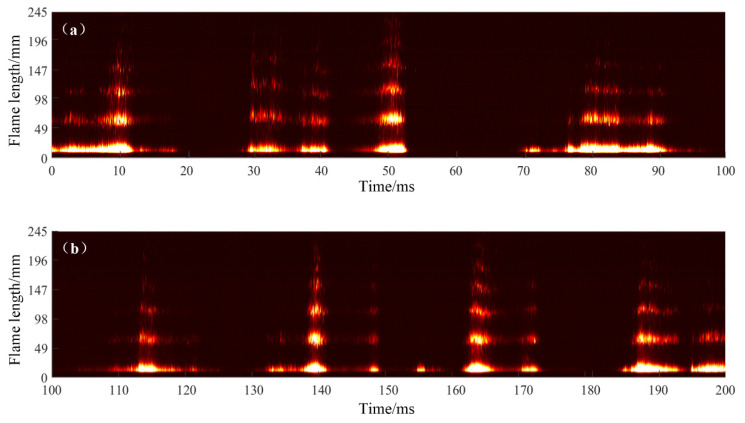
Pulsating combustion mode tail flame distribution over time (16–18). (**a**) Tail flame distribution over 0–100 ms, (**b**) tail flame distribution over 100–200 ms.

Two combustion modes, named stable combustion mode and pulsating combustion mode, can be distinguished clearly after processing the high-speed camera images under the successful initiation conditions. The exit flame of the resonant cavity is arranged along the time axis, with the length of the exit flame as longitudinal axis and time as the horizontal axis. Figure 5 shows the stable combustion mode. Although the length of the exhaust flame fluctuates in a certain range, the resonant cavity exit maintains the flame all the time without obvious interruption and extinction. For the pulsating combustion mode, the flame at the exit of the resonant cavity has obvious long time interruptions, which shows the process of flame extinction and combustion reorganization, as shown in Figure 6. Compared with the stable combustion mode, the flame brightness and length of pulsating combustion mode is brighter and larger, which indicates that the combustion in the resonant cavity is more intense.

**Figure 7:**
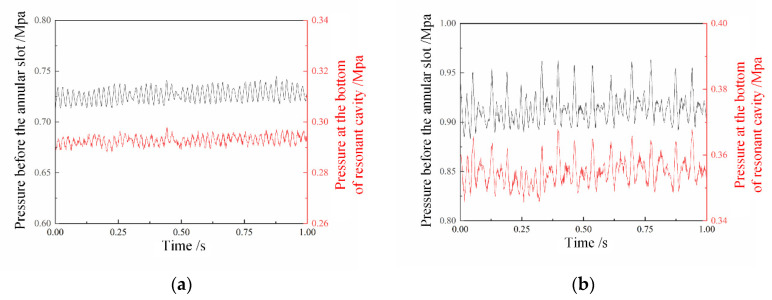
Pressure signals at the bottom of the resonant cavity and before the annular slot in two combustion modes. (**a**) Stable combustion pressure signal (15–18), (**b**) pulsating combustion pressure signal (16–18).

The pressure signal at the bottom of the resonant cavity and the injection pressure signal before the annular slot under the two combustion modes are shown in Figure 7. In the pulsation combustion mode, the pressure at the bottom of the resonant cavity is often higher than that in stable combustion mode, which means that strong shock wave focusing initiation events are happening in the cavity [17]. The injection pressure before the annular slot is also higher, which is the condition for the formation of a strong shock wave. Besides, the amplitude of injection pressure pulsation is larger, which can reach more than 0.6 bar. This means that a severe pressure back is happening in this combustion mode. The periodic initiation makes the pressure pulsation have a periodic law. Usually, when a strong shock wave focusing initiation happens, there will be several smaller amplitudes and weaker strength pressure fluctuations following. Relevant studies [31] have shown that high-pressure pulsation in the combustion chamber will hinder the recovery of fuel injection and affect the fuel filling degree [32], which will cause the next combustion intensity to decrease or even cause initiation failure, thus result in decreased pressure pulsation. From the high-speed imaging results of the pulsating combustion mode, it can be seen that one fierce combustion with a long flame tail is usually followed by a period of flameout, as shown in Figure 6. In the stable combustion mode, the injection pressure before the annular slot and the pressure at the bottom of resonant cavity are both low, which means there are soft ignition events with weak shock wave focusing [17]. There is no obvious high-pressure fluctuation, and the fluctuation amplitude is generally 0.1~0.2 bar, which means weak pressure back is occurring. In general, the comparison results show that the periodic initiation in the resonant cavity have a similar rule when the injection pressure changes, which can sufficiently indicate that the interaction between the combustion chamber and the supply plenum is the cause of the two combustion modes.

**Figure 8:**
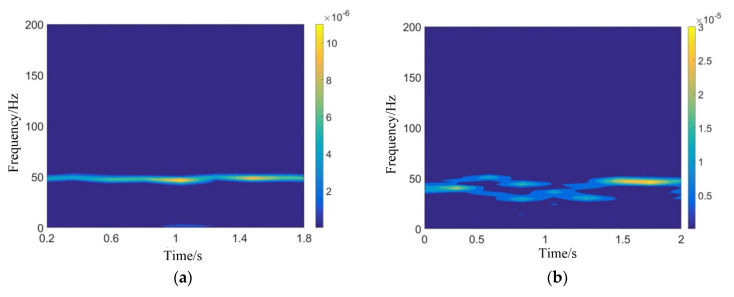
Time-frequency analysis of injection pressure before the annular slot in two combustion modes. (**a**) Stable combustion STFT result (15–18), (**b**) pulsating combustion STFT result (16–18).

STFT (short time Fourier transform) was performed on the injection pressure signals before the annular slot corresponding to the stable combustion mode and the pulsating combustion mode. As is shown in Figure 8, in the stable combustion mode, although there was always a flame at the exit of the resonant cavity, the time-frequency results show that the injection pressure fluctuation frequency was basically stable at about 48 Hz. The fluctuation amplitude was relatively small, and there was no sudden change or discontinuity. This shows that even in the stable combustion mode, there was periodic ignition behavior, resulting in small pressure pulsation caused by the soft ignition and weak pressure return, which will not lead to complete flameout. In the pulsating combustion mode, the pressure fluctuation frequency changed sharply in the range of 30~48 Hz (according to the STFT result), and the time-frequency signal interrupts from time to time. For the pulsating combustion mode, by counting the number of flame pulsations at the exit in Figure 7, it was found that the true flame oscillation frequency should be about 30~40 Hz. One possible reason accounting for this phenomenon is that, since there are still weak ignition events happening, accompanied by strong initiation events (as can be seen in Figure 6), it was difficult to distinguish them through the tail flame pictures. However, the statistic frequency in this paper still refers to the STFT average result.

Affected by the interaction of many complex factors, the evolution of shock wave focusing initiation and combustion often has inherent nonlinear characteristics. Traditional spectrum analysis methods often fail to reveal its nonlinear characteristics. Recently, nonlinear time series analysis methods have been used to study the dynamics of flow and combustion in combustion chambers of gas turbines and rotating detonation engines, especially in instances of combustion instability [33,34]. For the corresponding pressure-time signals in the two combustion modes, this paper used phase space reconstruction [35] and wavelet entropy diagram [36] methods to identify and distinguish them. Among them, the delay time of the phase reconstruction method was obtained by the autocorrelation method [37], the delay time was selected as 4, the embedding dimension was obtained by using the false nearest neighbor (FNN) method [38], and the embedding dimension was set to 6.

**Figure 9:**
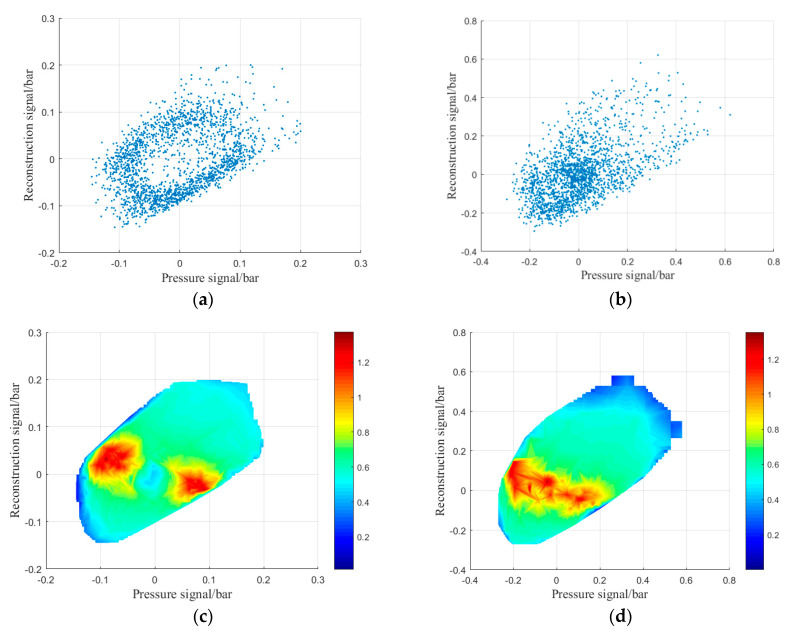
Phase space reconstruction diagram (**a**,**b**) and wavelet entropy diagram (**c**,**d**); (**a**,**c**) stable combustion mode, (**b**,**d**) pulsating combustion mode. (**a**) Phase space reconstruction diagram of stable combustion (15–18), (**b**) phase space reconstruction diagram of pulsating combustion (16–18), (**c**) wavelet entropy diagram of stable combustion, (**d**) wavelet entropy diagram of pulsating combustion.

As shown in Figure 9, the phase space reconstruction diagram and the wavelet entropy diagram corresponding to the pressure signals of stable combustion mode and pulsating combustion mode are different. In the stable combustion mode, the attractor structure behaves as an infinite cycle mode in phase space (Figure 9a). In the wavelet entropy diagram, the high wavelet entropy value is also symmetrically distributed in the region around the origin (Figure 9c). The reason for this phenomenon is that the pressure signal corresponding to the stable combustion mode has obvious periodicity, so the spatial points are distributed along the ring. Because of the large phase change at the signal peak, the high wavelet entropy usually appears at the high and low-pressure peak [39]. In the pulsating combustion mode, the attractor structure deviates slightly and fills in the inner region (Figure 9b), and the high wavelet entropy value distributes more evenly along the diagonal (Figure 9d). This is because combustion instability increases, and periodicity weakens in the pulsating combustion mode. Due to the large span of peak pressure distribution, the high entropy value of the wavelet is dispersed along the whole region.

### 3.3. Effects of Structural and Import Flow Parameters on Shock Focusing Combustion Characteristics

In this section, all experimental conditions are comprehensively analyzed, and the effects of different structural parameters and import flow conditions on shock wave focusing combustion characteristics are studied.

**Figure 10:**
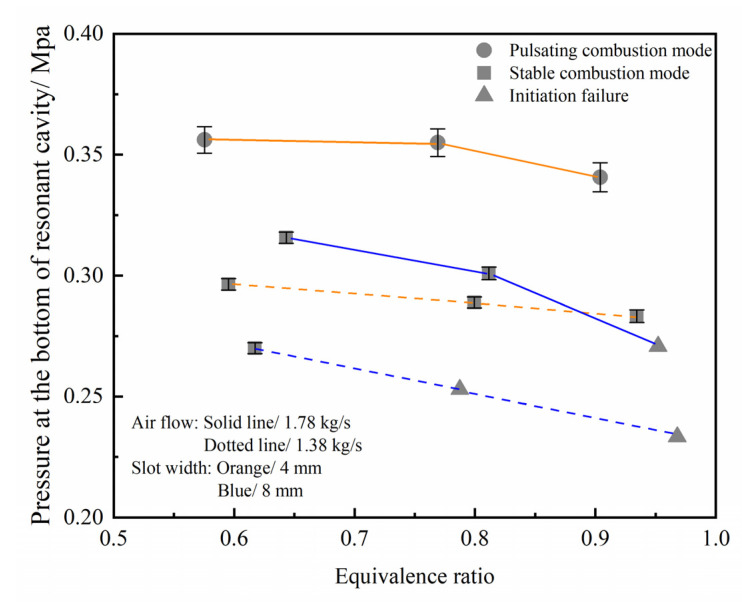
The pressure at the bottom of the resonant cavity and the combustion characteristics vary with the structure and import flow parameters.

The combustion modes under different working conditions were analyzed, as shown in Figure 10. It should be stated that the initiation failure mode had a violent irregular oscillation of the pressure baseline, thus the lowest pressure during the initiation process was defined as the pressure at the bottom of resonant cavity under the corresponding operation condition and the error bar was no longer marked. As can be seen in Figure 10, when the annular slot width was 8 mm, the initiation equivalence ratio boundary was narrow under low flow conditions, the successful initiation could only be achieved under fuel-lean condition. From the above analysis of the pre-combustion cracking part, it can be seen that the high equivalence ratio corresponds to the higher supplementary fuel ratio, where the cracking effect becomes worse and the initiation difficulty increases at this time. However, increasing the air flow can significantly widen the fuel-rich initiation boundary. When the width of the annular slot was 4 mm, successful initiation could be achieved under low flow conditions. This is because both the width of the annular slot and the import airflow affect the process of shock wave focusing and initiation by changing the pressure before the annular slot. According to the flow formula and shock wave focusing principle, reducing the width of the annular slot or increasing the air flow increases the upstream pressure of the annular slot, resulting in an increase in the pressure difference between the front and rear of the annular slot. The shock intensity formed by the high-speed airflow at the outlet of the annular slot also increases, which makes the pressure and temperature in the shock focusing area higher, and will increase the probability of successful initiation. When the annular slot width is too large or the inlet air flow is low, the shock wave intensity formed by the outlet airflow decreases, so that the combustible mixture can’t be initiated, which leads to the shock wave focusing initiation failure.

Further analysis shows that the stable combustion mode often occurs when the width and flow of the annular slot are relatively matched. That is, the airflow corresponding to the wide annular slot is larger, and the narrow annular slot usually corresponds to smaller airflow. In certain instances, the injection pressure before the slot was relatively low, and the shock wave intensity generated by the high-speed gas flow was relatively weak. This means that the high pressure generated by shock wave focusing initiation is too weak to influence injection recovery process, resulting in the dynamic balance between the pressure in the resonant cavity and the pressure before the annular slot. Thus, the combustion characteristic is the continuous and stable combustion characteristics. However, the pulsating combustion mode mainly occurs in the condition of a small annular slot width with a large airflow. The reason is that the shock wave intensity generated by the high-speed gas flow increases sharply due to the increase of the injection pressure before the annular slot, and the high pressure formed by the shock wave focusing combustion in the resonant cavity blocks the injection of gas flow in return, leading to combustion interruption. After the flame extinguishes and the high temperature and pressure combustion products are discharged from the resonant cavity, the pressure in the cavity gradually decreases. With the recovery of the injection mixture gas, the shock wave of the next cycle will refocus and initiate the fuel, and finally show strong pulsating combustion characteristics.

To obtain the relationship between the pressure at the bottom of the resonant cavity and the pressure before the annular slot under the combustion condition, the pressure recovery coefficient α is introduced to characterize the utilization efficiency of the pressure before the annular slot after the shock wave focusing. The formula is as follows:

(2) $$\alpha =\frac{P_{C}}{P_{0}}$$

**Figure 11:**
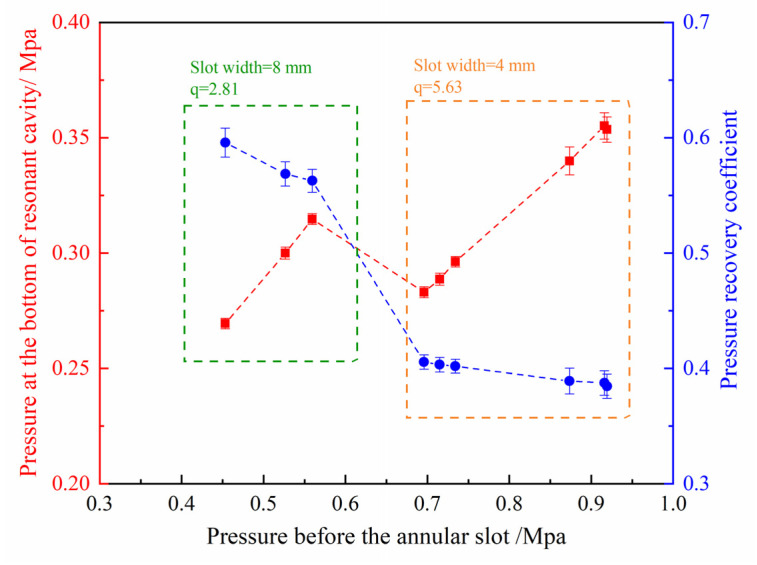
The pressure at the bottom of resonant cavity and the pressure recovery coefficient changes with the pressure before annular slot.

In Formula (2), $P_{C}$ (Mpa) represents the average pressure at the bottom of the resonant cavity, and $P_{0}$ (Mpa) represents the average pressure before the annular slot. Having summarized the pressure before the annular slot and the pressure at the bottom of the resonant cavity under all successful initiation conditions, the pressure recovery coefficient can be obtained, as shown in Figure 11. The left part (in the green frame) corresponds to the operational conditions with an 8 mm annular slot width. The right part (in the orange frame) corresponds to the operational conditions with a 4 mm annular slot width. With the increase of the pressure before the annular slot, the pressure at the bottom of the resonant cavity also increases, but the effective utilization rate of the pressure gradually decreases. The reason is that as the pressure before the annular slot increases, the intensity of the shock wave formed by the outlet airflow is greater, but the pressure loss is also greater. It can also be found from the figure that the pressure recovery coefficient corresponding to the larger annular slot width decreases faster with the increase of the pressure before the annular slot. The reason for this phenomenon is that the structural characteristic value corresponding to the 8 mm annular slot is 2.81. The change of inlet parameters has a greater impact on the parameters in the resonant cavity. The structural characteristic value corresponding to the 4 mm annular slot reaches 5.63, indicating that the injection size of the annular slot is far smaller than the size of the resonant cavity, and the pressure change before the annular slot has a weakened effect on the parameters in a resonant cavity, resulting in a slower decrease in the pressure recovery coefficient. However, the smaller slot width often has a greater injection pressure loss, due to the more serious degree of under expansion of air flow, which results in a lower pressure at the bottom of the resonant cavity, even though it has a higher injection pressure (as the sudden change on the cross-over regions in Figure 11 shows). Therefore, it is not advisable to set the injection pressure before the slot too high. It is necessary to ensure a better focus effect and maintain higher pressure utilization efficiency.

**Figure 12:**
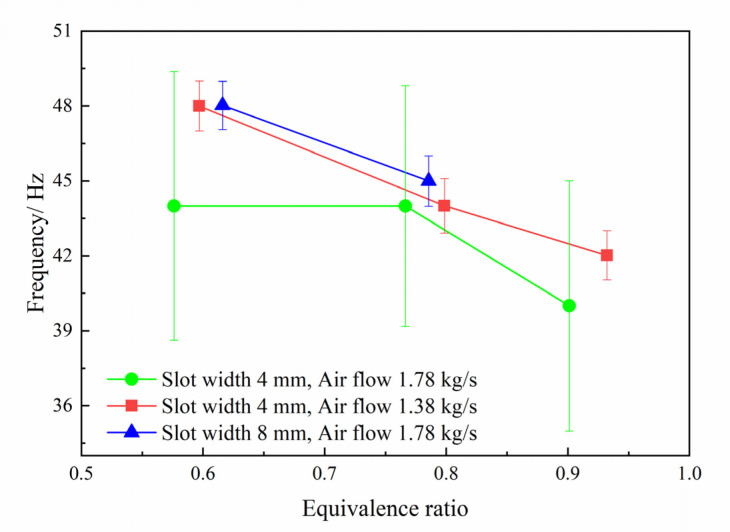
The frequency of pressure fluctuation changes with the equivalent ratio.

**Figure 13:**
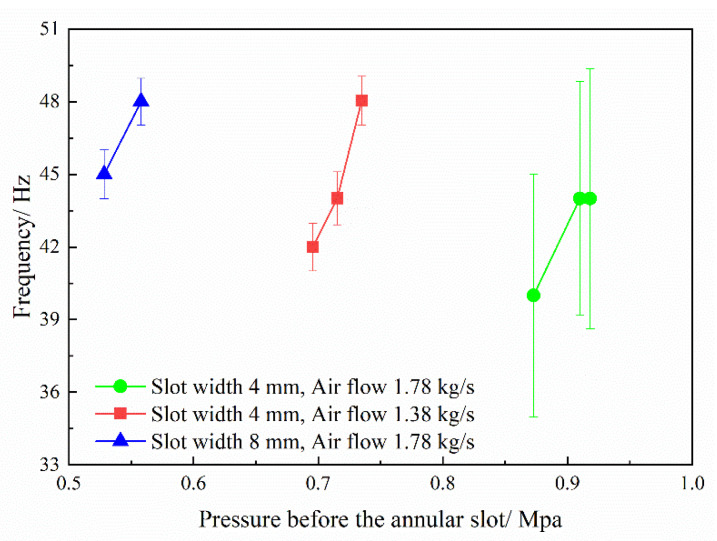
The frequency of pressure fluctuation changes with the injection pressure before the annular slot.

The pressure fluctuation frequency of resonant cavity under the condition of successful initiation is statistically analyzed, as shown in Figure 12 and Figure 13. With the decrease of the equivalence ratio, the pressure fluctuation frequency increases. According to the analysis above, the low equivalence ratio corresponds to the situation of the low supplementary fuel ratio. At this time, the cracking effect is better, and the chemical reaction activity of the combustible mixture is higher, which can speed up the shock wave focusing combustion cycle process, thereby increasing the frequency. Under the same structural parameters, increasing the pressure before annular injection can shorten the injection recovery time, thereby speeding up the combustion cycle. Therefore, the frequency of pressure fluctuation increases with the increase of the injection pressure. However, on the whole, the range of pressure fluctuation frequency change (for all operation conditions) is small, and mostly maintained between 38 Hz and 48 Hz. Considering the statistical frequency error, especially in the pulsating combustion mode, where the frequency error is about 5, reduces the credibility of the change trend. The above analysis shows that the equivalence ratio and injection pressure have a weak influence on the frequency.

Through examining the relevant literature [40,41,42] and further analysis, we have considered that possible reasons for the low pulsation frequency are: the structural characteristics of the combustion chamber, the high temperature and high pressure exhaust gas accumulated at the bottom of the resonant cavity that will form vortices, as well as the fact that that shedding of vortices needs to interact with the high-speed flow of fresh mixture, which leads to the slow exhaust process and effects the combustion cycle time. On the other hand, whether the pulsating combustion with large amplitude pressure oscillation is high-speed detonation combustion remains to be further verified by the following experiments.

### 3.4. Experimental Error Analysis

In this paper, the physical quantities directly measured or processed by the experimental measuring equipment included: pressure at the bottom of the resonant cavity, $P_{C}$, pressure before the annular slot, $P_{0}$ and the frequency of pressure fluctuation. The experimental error of these continuous signals of directly measured physical quantities over a period of time are usually expressed by standard deviation, as shown in Formula (3).

(3) $$\Delta =\sqrt{\frac{1}{n}\sum_{i=1}^{n}{\left(x_{i}-\bar{x}\right)}^{2}}$$

where $x_{i}$ is the real value of a certain physical quantity, $\bar{x}$ is the corresponding mean value averaged by time and Δ is the absolute error of this physical quantity.

The error of indirect quantity such as the pressure recovery coefficient α, which is obtained by the operation of other directly measured physical quantities, can be obtained through a transfer formula. Since α is the function of $P_{C}$ and $P_{0}$, that is $\alpha =f(P_{C},P_{0})$, the relative error of α ($\delta_{\alpha }$) and the absolute error of α ($\Delta_{\alpha }$) can be obtained by the following calculation:

(4) $$\delta_{\alpha }=\frac{\Delta_{\alpha }}{\alpha }=\sqrt{{\left(\frac{\partial \ln\alpha }{\partial P_{C}}\right)}^{2}\Delta_{P_{c}}^{2}+{\left(\frac{\partial \ln\alpha }{\partial P_{0}}\right)}^{2}\Delta_{P_{0}}^{2}}$$

(5) $$\Delta_{\alpha }=\alpha \delta_{\alpha }$$

## 4. Conclusions

In this paper, a shock wave focusing initiation engine was assembled and tested under a range of import flow parameters and structural parameters. Although the shock wave focusing combustion in the experiment results in detonation combustion remains to be further confirmed, this paper has found different combustion modes and combustion characteristics under various operational conditions. The results are concluded as follows:

(1) Due to the different matching relationship between the import air flow and the width of the annular slot, two combustion modes can be observed in the experiment, named the stable combustion mode and the pulsating combustion mode. Through image, time-frequency analysis, and nonlinear time series analysis methods, these two modes can be effectively distinguished. It is believed that the interaction between the resonant cavity combustion chamber and the supply plenum is the fundamental reason for the different combustion modes. The pulsating combustion mode can only be observed under the operation condition of small slot width (4 mm) with high air flow (1.78 kg/s).
(2) Within a certain range, a low supplementary fuel ratio can improve kerosene pyrolysis rate. High injection pressure before the annular slot can enhance the intensity of shock wave. Each can effectively increase the probability of successful shock wave focusing initiation. When the injection pressure is greater than 0.53 Mpa, the successful initiation equivalence ratio ranges from 0.6 to 0.95. When the injection pressure is lower than 0.45 Mpa, only the operation condition of 0.6 equivalence ratio can achieve successful initiation. From the perspective of pressure utilization efficiency, the injection pressure should not be too high. The highest pressure recovery coefficient can reach 0.595 under the operation condition of large slot width (8 mm) with low air flow (1.38 kg/s).
(3) The low equivalence ratio and high injection pressure can shorten the combustion cycle and increase the frequency of combustion pressure fluctuation. Generally speaking, the influence scope of the above parameters is limited. The fluctuation frequency is maintained between 38 and 48 Hz.

## Figures and Tables

**Table 1:** Experimental conditions of shock wave focusing initiation.

Operational Condition Serial Number	Experiment Serial Number	Slot Width/mm	Air Flow/ (kg∙s^−1^)	Kerosene Flow/ (g∙s^−1^)	Supplementary Fuel Ratio	Equivalence Ratio
Con. 1	15–18	4	1.38	56.0	5.49	0.60
Con. 2	15–20	4	1.38	75.0	7.35	0.80
Con. 3	15–22	4	1.35	85.0	9.55	0.93
Con. 4	16–04	4	1.80	70.7	7.81	0.58
Con. 5	16–18	4	1.84	96.0	10.65	0.77
Con. 6	16–29	4	1.83	112.0	12.73	0.90
Con. 7	12–44	8	1.38	57.8	5.58	0.62
Con. 8	12–45	8	1.39	74.3	7.10	0.79
Con. 9	12–47	8	1.40	91.6	8.64	0.97
Con. 10	12–56	8	1.78	78.0	7.50	0.64
Con. 11	12–58	8	1.78	98.2	9.35	0.81
Con. 12	12–59	8	1.77	114.5	11.08	0.95

**Table 2:** The cell size and critical ignition energy data of gas fuel (initial pressure ≈ 101.3 kPa, initial temperature ≈ 293 K, and equivalence ratio ≈ 1) [29].

Species	Cell Width/mm	Critical Initiation Energy/J
H_2_	8.0~15.1	4248.8~9040.0
CO + 2.9%H_2_	32	
CO + 8.69%H_2_	16.9	
C_2_H_2_	4.6~9.2	4332.0~6780.0
CO + 1.3%C2H_2_	29.9	
CO + 3.71%C_2_H_2_	10.9	
C_2_H_4_	19.5~33.8	55,596.0~60,568.0
CO + 2.14%C_2_H_4_	33.8	
CO + 4.23%C_2_H_4_	25.2	
CH_4_	279.6~349.5	88,658,800.0
